# Deciphering Protein Glycosylation by Computational Integration of On-chip Profiling, Glycan-array Data, and Mass Spectrometry*[Fn FN2]

**DOI:** 10.1074/mcp.RA118.000906

**Published:** 2018-09-26

**Authors:** Zachary Klamer, Peter Hsueh, David Ayala-Talavera, Brian Haab

**Affiliations:** From the Van Andel Research Institute, 333 Bostwick NE, Grand Rapids, MI 49503

**Keywords:** Glycoproteomics, Glycosylation, Protein Array, Micro Arrays, Bioinformatics, Exoglycosidase, Glycosidase

## Abstract

The difficulty in uncovering detailed information about protein glycosylation stems from the complexity of glycans and the large amount of material needed for the experiments. Here we report a method that gives information on the isomeric variants of glycans in a format compatible with analyzing low-abundance proteins. On-chip glycan modification and probing (on-chip gmap) uses sequential and parallel rounds of exoglycosidase cleavage and lectin profiling of microspots of proteins, together with algorithms that incorporate glycan-array analyses and information from mass spectrometry, when available, to computationally interpret the data. In tests on control proteins with simple or complex glycosylation, on-chip gmap accurately characterized the relative proportions of core types and terminal features of glycans. Subterminal features (monosaccharides and linkages under a terminal monosaccharide) were accurately probed using a rationally designed sequence of lectin and exoglycosidase incubations. The integration of mass information further improved accuracy in each case. An alternative use of on-chip gmap was to complement the mass spectrometry analysis of detached glycans by specifying the isomers that comprise the glycans identified by mass spectrometry. On-chip gmap provides the potential for detailed studies of glycosylation in a format compatible with clinical specimens or other low-abundance sources.

## Abstract (precis)

A new method is presented to decipher information about the isomeric variants of the glycans attached to purified proteins. The method takes advantage of the specificities of glycosidases and lectins, combined with computational integration of the data with information from glycan arrays and mass spectrometry. The application to microspots of proteins makes it compatible with analyzing low-abundance proteins, providing the potential for studying proteins derived from clinical specimens.

## Graphical Abstract

**Figure:**
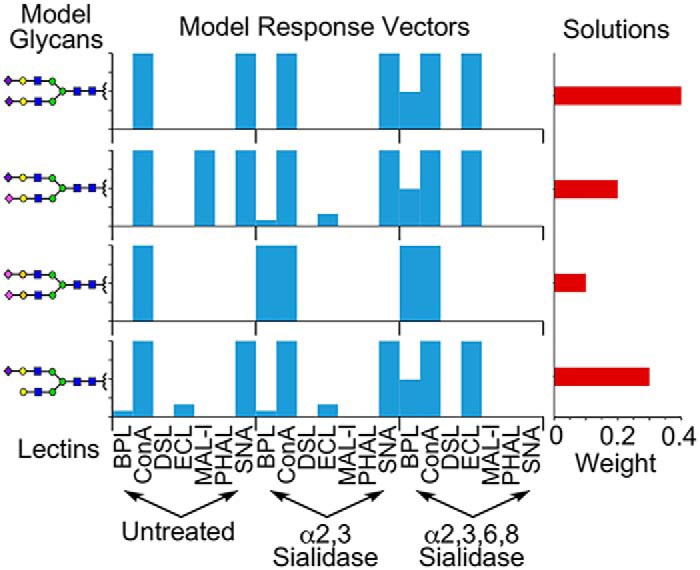

**Highlights**

- Method to probe the isomeric variants of the glycans attached to purified proteins.
- Uses multiple rounds of glycosidase cleavage and lectin profiling.
- Computation integration of lectin-binding, glycan-array, and mass spectrometry data.
- Applied to microspots for compatibility with analyzing low-abundance proteins.

Obtaining detailed information about glycans on proteins is experimentally difficult. The difficulty in part stems from the complex nature of glycosylation. Because glycans are not simply linear polymers, as are proteins and nucleic acids, but can be branched at multiple points and have multiple options in the sequence of monosaccharides, the reducing-carbon linkage (3′, 4′, etc.), and anomeric type of linkage (alpha or beta), a given set of monosaccharides composing a glycan may have many possible isomers. Mass spectrometry (MS)^1^ is widely used to study glycans that are released from the protein backbone, and it readily provides information on the type and number of monosaccharides (1), but the mass does not provide information about the branching, sequence, linkages, or anomeric state. The options are constrained according to allowed biosynthetic pathways, but even within the allowed options, many isomers can exist. Information about sequences and linkages can be acquired through additional methods such as MS^n^ fragmentation (2), chemical modifications (3), exoglycosidases digestions (2), ion mobility separation (4), nuclear magnetic resonance imaging (5), or inferences based on biosynthetic pathways. Such methods require specialized tools, increased sample amounts, and expert knowledge. Integrating orthogonal data types can help resolve ambiguities from any single data source, yet the integrations are done manually and qualitatively; computational means of linking disparate data have not appeared.

Another challenge is obtaining enough material for a detailed analysis. The sample requirements of the various MS-based approaches are being reduced regularly, but at present, sample amounts can be a problem when analyzing a protein from a clinical specimen or a model system with limited amounts of the protein available. To analyze the glycan structures of a purified protein, a common approach is detach the glycans, purify them, and analyze them by MS/MS. Such methods require 5–10 μg of a purified protein to acquire data on the masses and relative abundances of the glycans. With more sample and specialized methods, information can be obtained about the sequence, linkage, and anomeric variants of specific features (6, 7). A related approach is to proteolytically digest a purified protein and analyze the masses of the glycopeptides. Such a method is valuable for locating glycosites and quantifying relative site occupancies, but it is not as effective as detached glycan analysis for analyzing whole glycans. Glycopeptide analysis by MS can be quite sensitive, requiring only 100–200 ng of purified protein per injection, although obtaining enough for the digestions and purifications can be challenging in certain cases. A basic analysis of some features of glycosylation can be acquired using 1 μg or less of purified proteins. For example, lectin blotting of gel-separated prostate-specific antigen detected a specific glycoform using only 100 ng of protein (8), and *in situ* glycan release and analysis by MALDI MS could be compatible with similarly low protein quantities (9). Thorough analyses of the sequences, branching, linkages, and anomeric states of protein glycosylation are typically limited to biopharmaceutical applications where material can be obtained in high abundance or from recombinant production, as in a study of erythropoietin (10). Additional methods to complement the existing toolbox of glycan-analysis methods would be useful, especially if they could provide increased information about sequences, branching, linkages, and anomeric states, while being compatible with application to clinical specimens.

We previously introduced an on-chip method that can provide information about the glycosylation of proteins using a small amount of input material (11). The method, called on-chip glycan modification and probing (on-chip gmap), involves the on-chip capture of proteins out of clinical samples or the direct printing of a purified protein, the repeated modification of the glycans using exoglycosidases, and the probing of the resulting glycans using panels of lectins. The method is analogous to conventional glycan sequencing by the parallel or sequential application of exoglycosidases and analysis by electrophoresis (12), but it analyzes the cleaved glycans by lectin profiling rather than mobility shifts. We demonstrated that the method can help to meet the need of increased information about isomeric variants while having low sample requirements.

The previous work demonstrated the feasibility of the approach, but it also revealed areas that needed development. The analysis algorithms were built around solving for glycan motifs, or patterns of substructures, rather than complete glycans. This method left uncertainty in the arrangements of the motifs with respect to each other and uncertainty in which motifs were part of the same glycan. As a result, the method also left uncertainty about the heterogeneity in glycosylation that is present on nearly every glycoprotein because information was not given about whether the individual motifs comprised few or many glycans. Furthermore, based on the potential for improved accuracy by integrating orthogonal data types, we needed a way to computationally integrate the on-chip-gmap data with analyses from glycan arrays, which give details about lectin and exoglycosidases specificity, and from MS experiments, which provide the monosaccharide compositions of the glycans.

In this work, we developed an algorithm to process data from on-chip gmap in a way that addresses the above limitations: It interprets the heterogeneity and relative abundances of glycans in a sample, and it computationally integrates information from glycan-array and MS experiments. To test the system, we analyzed glycoproteins for which detailed information on glycosylation was obtainable, meaning that they could be acquired in high abundance. For working out the method, we printed microspots of proteins directly on the slide, as opposed to capturing the proteins out of solution using microspots of an antibody (11). We compared the independently determined information with results from on-chip gmap, either alone or in combination with MS. We show that on-chip gmap provides accurate information about glycosylation and that the integration of basic mass information provides further improved accuracy. In addition, we show that on-chip gmap promises to be valuable for assisting the interpretation of MS data, in particular by specifying the isomers that comprise each glycan.

## EXPERIMENTAL PROCEDURES

### 

#### 

##### Protein Microarray Fabrication and Use

The lectins, control proteins, and enzymes were purchased from various sources (Table I). We printed the glycoproteins onto coated microscope slides (PATH, Grace Bio-Labs, Bend, OR) using a robotic arrayer (2470, Aushon Biosystems, Billerica, MA). The arrayer spotted ∼340 pl of a 250 μg/ml solution per spot (0.085 ng per spot), and six replicate spots per protein, resulting in 0.5 ng/array. We used about 66 μl of protein solution to load the source plate, based on 12 pins and ∼5 μl/well. Each slide contained 48 identical arrays arranged in a 4 × 12 grid with 4.5-mm spacing between arrays, and each array had the same proteins printed with six replicates. After printing, hydrophobic borders were imprinted onto the slides (SlideImprinter, The Gel Company, San Francisco, CA) to segregate the arrays and allow for multiple, separate sample incubations on each slide (13).

The assays were modified from the protocol described previously (14). We prepared α2–3 neuraminidase (P0728L, New England Biolabs, Ipswich, MA) or α2–3, 6, 8 neuraminidase (P0720S, New England Biolabs) at 250 U/ml in the supplied reaction buffer and incubated each separately on arrays containing the spotted glycoproteins overnight at 37 °C. We prepared the β1–3 galactosidase (P0726S, New England Biolabs) or β1–4 galactosidase (GKX-5014, Prozyme, Hayward, CA) enzymes at a 1:100 dilution in the supplied reaction buffers and incubated them separately on arrays overnight at 37 °C. The arrays not treated with enzymes were incubated with the reaction buffers in the same conditions. The slides were washed three times for 5 min each in 1X phosphate-buffered saline containing 0.1% Tween-20 and spin-dried. We incubated each array with 6 μl of a biotinylated lectin solution (3 μg/ml in 1X PBS with 0.1% Tween-20 and 0.1% BSA) for 1 h. We washed and dried the slides as above, incubated Cy5-conjugated streptavidin (43–4316, Invitrogen, Carlsbad, CA) (2 μg/ml in the same buffer as the lectins) for 1 h, and performed a final wash and dry step. The slides were scanned for fluorescence at 633 nm using a microarray scanner (InnoScan 1100 AL, Innopsys, Carbonne, France).

The fluorescence images were quantified and analyzed using custom, in-house software (15). The software subtracts the local, mean background from the median signal of each microspot, averages replicate spots from each array, and then averages equivalent data across replicate arrays.

##### Calculations

We used Matlab (R2017a, Mathworks, Natick, MA) to develop software, called GlycanSolver, to process on-chip-gmap data and calculate the relative abundances of glycans. The formation of response vectors, which are the predicted patterns of lectin binding to model glycans in each round of enzymatic modification, was based on glycan-array data from the Consortium for Functional Glycomics. Using the MotifFinder software presented earlier (16), we defined the glycan motifs that each lectin binds, selected orthogonal sets of motifs that cover the main types of binding, and calculated a “binding score” for each motif based on the average binding to glycans containing the motif relative to control glycans.

To define the modifications to model glycans upon enzymatic treatment, we used the method presented earlier (16) in which we define recognition and replace motifs for each enzyme. If the recognition motif is present, the software substitutes the recognition motif with the replace motif to simulate context-sensitive enzyme activity. To predict lectin binding, the MotifFinder software searches the unmodified and modified model glycans for the motifs defined for each lectin, and where a motif is present it uses the binding score to approximate the binding level. Given that a lectin's affinity is not equivalent for all motifs, the binding scores allow us to take advantage of observed gradations in lectin binding to glycan arrays when building the model response vectors. Both the observed on-chip gmap data and the model response vectors are normalized by dividing all data for a given lectin by the highest observed binding (or predicted binding to model glycans) for the lectin. To organize the model glycans by similarity in lectin-binding patterns, the model response vectors were clustered (hierarchical clustering by Euclidean distance with complete linkages) and grouped by shared subclusters at a uniform cutoff in distance between clusters.

The next step was to use linear algebra to fit the modeled response vectors to the observed response vector. The glycan-prediction weights are the nonnegative least squares solution to the equation

$$\min{\left\|Ca-d\right\|}_{2}^{2}where\;a\;\geq \;0$$

where C is an N × M matrix of M glycans and N lectin-binding predictions, and *d* is an N × 1 vector of observed lectin binding. The result, *a*, is an M × 1 vector of glycan weights. The nonnegative constraint restricted the glycan prediction weights to positive values and allowed the interpretation of the weights as relative abundances.

To reduce the impact of colinearity (model glycans with identical response vectors or that are linear combinations of each other), we filtered the input glycan models to select a single representative from each perfectly colinear set of glycans (using the glycan with the most monosaccharides evaluated by the lectins). To compensate for remaining colinearity and to reduce the overemphasis of a particular solution, we collect samples of alternative fits by repeatedly excluding one of the initially predicted glycans and refitting the model. If a resulting model has an R^2^ within 0.05 of the initial model and uses no more than two additional glycans, we consider it an alternative. We recursively sample the alternative models, with limitations proportional to the depth of the recursion. The total number of samples collected is 100 times the number of glycans in the initial model so that sampling grows proportionally with model complexity. Glycans included in at least 10% of the fits are included in the final report, and the reported weights are the averages over all of the acceptable fits.

## RESULTS

### 

#### 

##### Deconvoluting Glycans Probed with a Sequence of Lectins and Enzymatic Modifications

**Fig. 1.**
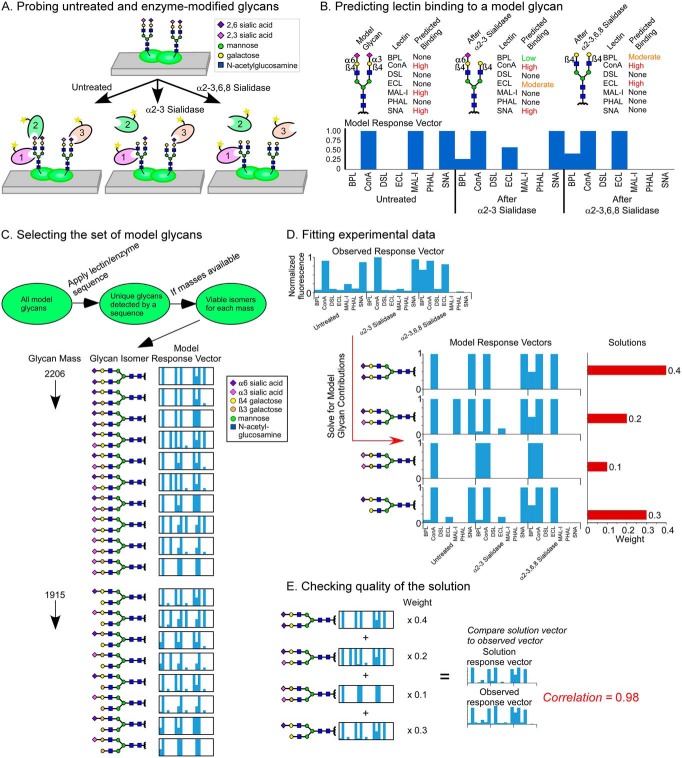
**Solving for glycan structures using patterns of lectin binding.** (*A*) Microspots of proteins are probed by a panel of lectins, applied with or without modification of the glycans using exoglycosidases. (*B*) Lectin binding to model glycans is simulated for each round of profiling. The predicted binding levels are assembled into a response vector. (*C*) Sets of model glycans are defined that represent potential solutions to the glycans in a sample. The set can be filtered to include only those that are tested by the lectins and enzymes used in the experiment or to include only those that have masses known to be present. A model response vector is produced for every model glycan in the final set. (*D*) We use linear algebra to solve for the combinations of model response vectors that give a least-squares fit of data. Any number of glycans from the input list may be included in a fit; in this example data, the least squares fit uses four model response vectors from the larger set in 1C. (*E*) We multiply each model response vector in the solution by its weight and then sum the results to produce a solution response vector. The solution response vector is compared with the observed response vector to assess the closeness of the fit.

The experimental data are collected by a method we introduced earlier (11) (Fig. 1*A*). Microspots of glycoproteins are immobilized on a microscope slide, and in parallel arrays, the proteins are probed with a series of lectins with or without prior treatment with exoglycosidases to expose underlying features.

The analysis begins with defining model glycans that represent potential solutions to the glycans that are present in the sample. For each model glycan, we predict the level of binding of each lectin to the untreated glycan and to each enzyme-modified glycan (Fig. 1*B*). The predictions are done using a method introduced earlier (16), which uses data from glycan arrays to define the binding by each lectin to various glycan motifs and then searches for the motifs on the model glycans. The results of these predictions are “model response vectors” showing the pattern of binding across the lectins and conditions.

We then select a set of glycans representing potential solutions to the glycans in the sample (Fig. 1*C*). The list would include any glycans that could be probed by the sequence of lectins and enzymes, constrained by other information. If the masses of the glycans are available from MS, the user would use only viable isomers of the masses (Fig. 1*C*). For each glycan in the set, we determine the model response vector.

Next, we determine which of the model response vectors could contribute to the observed, experimental response vector. Because most glycoproteins have a mix of glycans, we use linear algebra to solve for the best combinations of model response vectors that make up the observed response vector (Fig. 1*D*). The weight of each vector is the multiplier that indicates its contribution to the experimental response vector, which in principle scales with the relative abundance of the glycan. Because any particular fit might not represent the only reasonable solution, we iteratively remove one of the predicted glycans and repeat the fits 100 times in each iteration so that we sample the fits in proportion to glycoprotein complexity and build up statistics on which glycans are most frequently selected. The final output (Table S1) reports the glycans present in >10% of the fits, along with various statistics for each glycan, such as the weight averaged over the fits. To assess the goodness of the fit, we compare the observed response vector to the solution response vector, which is the sum of the model response vectors after multiplication of each by their weights (Fig. 1*E*).

##### Reference Standards

To test the system, we used glycoproteins that could be obtained in high abundance. Such glycoproteins would allow analyses of glycosylation using established technologies that require large amounts. We then could compare the resulting information with the output from on-chip gmap. We selected a protein with relatively simple glycosylation, human transferrin, and a protein with complex glycosylation, bovine fetuin.

**Fig. 2.**
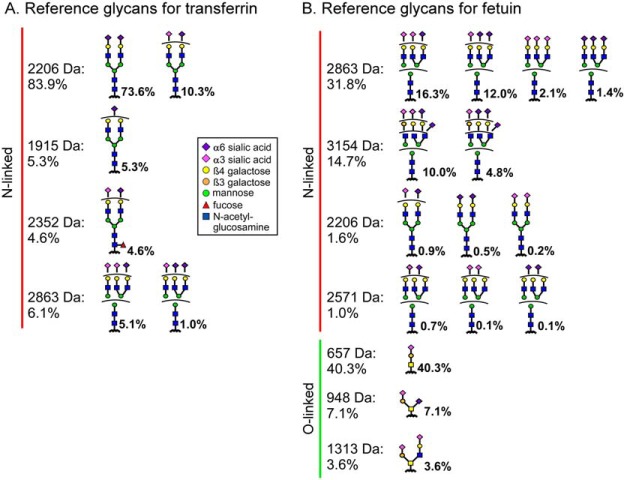
**Reference glycans.** The reference glycans were culled from independent sources taken to be a reliable, independent standard for what is present on transferrin and fetuin. Information was integrated from MS, capillary electrophoresis, and published data for (*A*) transferrin and (*B*) fetuin. Ambiguity in branch arrangements is indicated by a curved bar.

For transferrin, we relied on our data from earlier (11), which used the same source of material (Table I) as used here. The sialic acid linkage information had been determined through the ethyl esterification of terminal sialic acid residues, as adapted from Reiding *et al.* (3) and as previously reported (17). The dominant glycan of transferrin at 2,206 Da is a di-sialylated, biantennary glycan, mostly with both sialic acids in the α2–6 position but some with one α2–6 and one α2–3 (Fig. 2*A* and Table S2). The assignment of fucose in the glycan at 2,252 Da to the core position rather than on an outer arm was based on our previous chromatography data (11) using the same material as used here. Others have seen outer-arm fucose on transferrin (18), but several other studies failed to see it, perhaps due to actual differences in the sample.

**Table I:** Biological reagents

Name	Type	Source	Catalog #
*Maackia amurensis* lectin I (MAL-I)	Lectin	Vector Laboratories	B-1335
*Sambuca nigra* agglutinin (SNA)	Lectin	EY Labs	BA-6802-1
*Erythrina cristagall*i lectin (ECL)	Lectin	Vector Laboratories	l-1140
*Bauhinea purpurea* lectin (BPL)	Lectin	Vector Laboratories	B-1285
Concanavalin A (ConA)	Lectin	Vector Laboratories	B-1005
Phytohemagglutinin L (PHAL)	Lectin	Vector Laboratories	B-1115
*Datura stramonium* lectin (DSL)	Lectin	Vector Laboratories	BK-3000
*Maackia amurensis* lectin II (MAL-II)	Lectin	Vector Laboratories	B-1265
*Griffonia simplicifolia* lectin II (GSL-II)	Lectin	Vector Laboratories	B-1215
*Sclerotium rolfsii* lectin (SRL)	Lectin	Wako	199-17271
Laminin, human	Purified glycoprotein	Sigma Aldrich	l-6274
Transferrin, human	Purified glycoprotein	Fitzgerald	30C-CP4060
Fetuin, bovine	Purified glycoprotein	Sigma Aldrich	F2379
Haptoglobin, human	Purified glycoprotein	Calbiochem	372022-1MG
α2–3 Neuraminidase (sialidase)	Enzyme	New England BioLabs	P0728L
α2–3, 6, 8 Neuraminidase (sialidase)	Enzyme	New England BioLabs	P0720S
PNGase F	Enzyme	New England BioLabs	P0704S
β(1–3) Galactosidase	Enzyme	New England BioLabs	P0726S
β(1–4) Galactosidase	Enzyme	Prozyme	GKX-5014

For fetuin, we integrated newly acquired data using MS (available as Supplementary Data) and capillary electrophoresis (performed at the University of Georgia (6)) with published results (19, 20). The full Fourier-transform mass spectrometry (FTMS) spectra of the *N*-linked glycans showed a broad distribution among triantennary and biantennary structures (Fig. S1). The relative abundances among the glycans (Fig. 2*B* and Table S2) agreed well with previous analyses at the University of Georgia (Table S3), supporting the consistency of the protein preparations and analyses. The sialic acid linkages were probed by fragmentation analysis in MS^n^ (Fig. S2), which showed a mixture of α2–3 and α2–6 for each mass. Information on the galactose linkages of the top masses was obtained by retention times in capillary electrophoresis (Fig. S3 and Table S4). The galactose linkage is mainly β1–4 on all masses, except it is about 10% β1–3 on a single arm of the triantennary glycans. To obtain further detail about the linkages, we used published NMR data (19), which provided information on the relative abundances of the isomers with α2–3 *versus* α2–6 sialic acid or β1–3 *versus* β1–4 galactose but not exact branch arrangement of the linkages.

Fetuin also contains a substantial amount of O-linked glycans. The relative amounts between *N*-linked and O-linked glycans, and the relative amounts within O-linked glycans, were based on a previous study (20) that was in agreement with previous analyses performed at the University of Georgia (data not shown).

These analyses highlight the prevailing difficulties in analyzing protein glycosylation since ambiguity remained even after integrating all available information. In particular, the numbers of sialic acids with α2–3 or α2–6 linkage or of galactose with β1–3 or β1–4 linkage could be estimated, but the specific branch arrangements could not be determined from the available data (Fig. 2).

##### Analysis of Glycosylation on the Standard Proteins

**Fig. 3.**
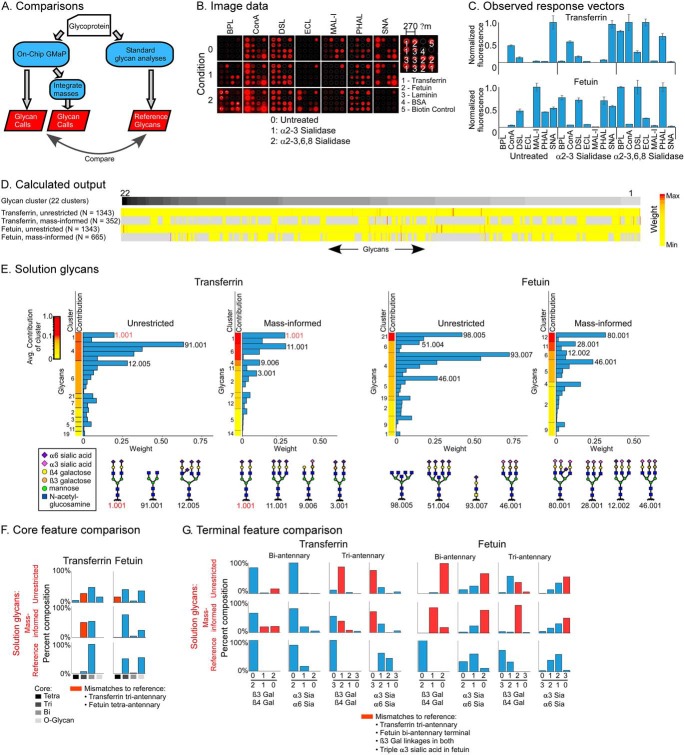
**Solving for glycans on transferrin and fetuin.** (*A*) We made glycan determinations using on-chip gmap alone or with integration of information on the masses present in each sample. The glycan calls were compared with the reference glycans. (*B*) Raw image data. (*C*) Observed response vectors. (*D*) The unrestricted list included 1,343 glycans grouped into 22 clusters based on similarity of response vectors. The color scale indicates the calculated weight of each glycan. The mass-informed input lists were subsets of the unrestricted lists, with gray indicating not-included glycans. (*E*) The glycans in the solution of each analysis were grouped by cluster and ordered by the average contribution to the response vector of the glycans in the cluster. Structures are given for the top glycan from each cluster that contributes at least 0.05 (5%) of the total. The red text indicates glycans present in the reference. (*F*, *G*) The fraction of glycans containing a particular core (panel *F*) or terminal feature (panel *G*) was determined in the reference glycans and in the two sets of solution glycans. (The α6 sialic-acid count includes a small number of glycans that lack one sialic acid.) The red columns indicate discrepancies between the reference and the solutions.

To assess the performance of the system, we made calls on the glycans both with and without integration of glycan masses, and we compared the calls to the reference glycans (Fig. 3*A*). In the integration of MS data, we used only the masses—the compositions of monosaccharides—and not additional information from fragmentation or chromatography.

On-chip gmap was run with seven different lectins on arrays that were untreated or treated with α2,3-sialidase or α2,3,6,8-sialidase. This sequence of lectins and enzymes was predicted to provide information about the relative amounts of 2,3-linked and 2,6-linked sialic acids and some information about underlying structures. The microarray images (Fig. 3*B*) showed major changes in binding consistent with the known specificities of the enzymes and lectins, and the response vectors of transferrin and fetuin were very different from each other (Fig. 3*C*). The complete data are given in Table S5.

This experiment consumed about 32 ng of each protein (0.5 ng/array × 3 arrays/condition × 3 conditions × 7 lectins). To study proteins from biological samples, one would spot antibodies and capture the proteins out of an incubated sample, as we demonstrated in the analysis of MUC5AC glycosylation (11). But in this research, to work out the methods and because we did not have good antibodies against fetuin, we directly spotted the purified proteins. The amount of material used to load the source plates for printing was much more than consumed—we used a 12-pin contact printer for a total of 66 μl (∼5 μl/pin) of a 250 μg/ml solution (16.6 μg of protein)—but the remaining material could be recovered for later use.

The calculations using the unrestricted set of 1,343 glycans—not restricted to certain masses or types of glycans—pulled out 22 glycans for transferrin and a different set of 26 glycans for fetuin (Fig. 3*D* and Table S1). When restricting the input to glycans with masses found in the glycoproteins—352 glycans for transferrin and 665 glycans for fetuin—the analyses resulted in 19 glycans for transferrin and 23 for fetuin, with little overlap in output between the analyses (Fig. 3*D* and Table S1). The input glycans (Table S6) were grouped into 22 clusters according to the similarity of their vectors because highly similar vectors potentially represent alternate solutions. The solution response vectors (Fig. S4) showed good correlations (0.92–0.96) with the observed response vector for the solutions found with or without mass information.

To view the weights and confidences of individual solutions, the glycans were grouped into their clusters and ranked by the average contribution to the solution response vector of the glycans in the cluster (Fig. 3*E*). The contribution is the relative amount of the observed response vector explained by the glycan's model response vector and serves a measure of confidence. The highest-confidence solution glycans generally match features in the reference glycans, such as glycan 1.001 matching the most-abundant reference glycan for transferrin. The solutions produced using the mass-informed set eliminated incorrect answers, such as a partially elongated glycan (91.001) in transferrin and a tetraantennary glycan (98.005) in fetuin, indicating the value of integrating the mass information.

Although the experiment was not designed to probe core types, we examined whether such information nevertheless was achieved. Using the two sets of solution glycans—from the unrestricted and the mass-informed analyses—as well as the reference glycans, we summed the percentages of each core type (Fig. 3*F*). The comparison showed general agreement but some differences. Triantennary glycans were overrepresented for transferrin and tetraantennary glycans were overrepresented for fetuin, although incorporating mass information minimized that result. We performed a similar analysis of the proportions of specific terminal features on the biantennary or the triantennary glycans (Fig. 3*G*). For both transferrin and fetuin, the proportions of β1–3 linkages were overrepresented, even in the mass-informed analyses. The references glycans for transferrin contain none of this linkage, and the reference glycans for fetuin contain a small amount on the triantennary glycans. Likewise, the proportion of α2–3 *versus* α2–6 sialylation was overrepresented for fetuin and the triantennary glycans of transferrin. Thus, the calculations found solutions that generally agreed with the reference glycans, but the solutions differed in specific features such as the branching in transferrin and the proportions of galactose β1–3 and sialic acid α2–3 linkages in both proteins.

##### Targeting Specific Structures on a Complex Glycoprotein

**Fig. 4.**
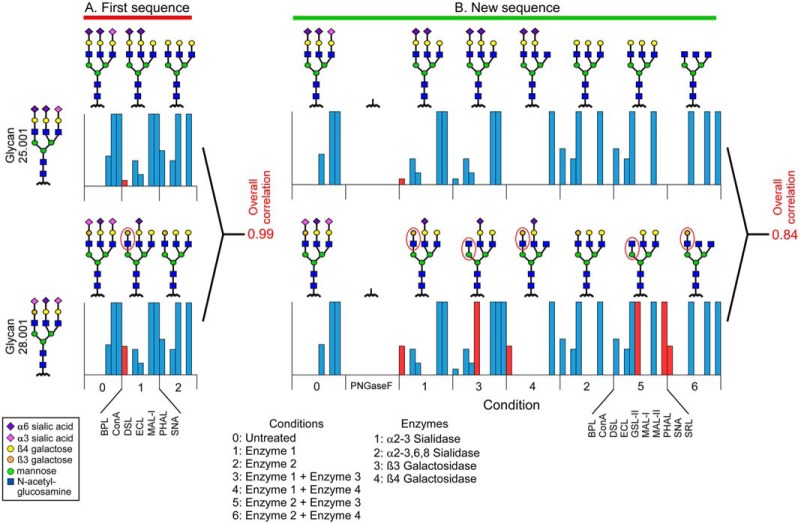
**Designing lectin and enzyme sequences to differentiate specific features.** We compared the response vectors of two tri-antennary glycans that differed only in the sialic acid and galactose linkages of one arm. The structures and response vectors are shown for each round of enzymatic modification and lectin profiling in (*A*) the first experiment and (*B*) a newly designed experiment. The red ovals indicate features that would be differentially bound, and the red columns indicate the lectin binding that is predicted to distinguish the glycans.

The areas of discrepancy were explainable based on the sequence of lectins and enzymes used. The sequence did not thoroughly probe the underlying features of the glycans, including the galactose linkages but, rather, mostly the termini. We therefore explored whether we could design an experiment that in theory would provide greater discrimination of underlying features, including the branching and the galactose linkages. In the solution of fetuin, glycans 25.001 and 28.001 from cluster 11 were both viable solutions for fetuin, but they differed in the sialic acid and galactose linkages on one arm. Their predicted response vectors were essentially indistinguishable (correlation = 0.99) using the lectins and enzymes of the first experiment (Fig. 4*A*). We then designed an experiment incorporating galactosidases with differing specificities toward β1–3 and β1–4 galactose, used in combination with the two sialidases used in the first experiment, and lectins chosen to probe the exposed features. We found that the response vectors of glycans 25.001 and 28.001 were now distinguishable, with a lower correlation of 0.84 (Fig. 4*B*). The distinction arose mainly from lectins—Bauhinea purpurea lectin (BPL), Griffonia simplicifolia lectin (GSL), and Sclerotium rolfsii lecti (SRL)—that bound to galactose or N-acetyl-glucosamine (GlcNAc) as they were differentially exposed by the enzymes.

**Fig. 5.**
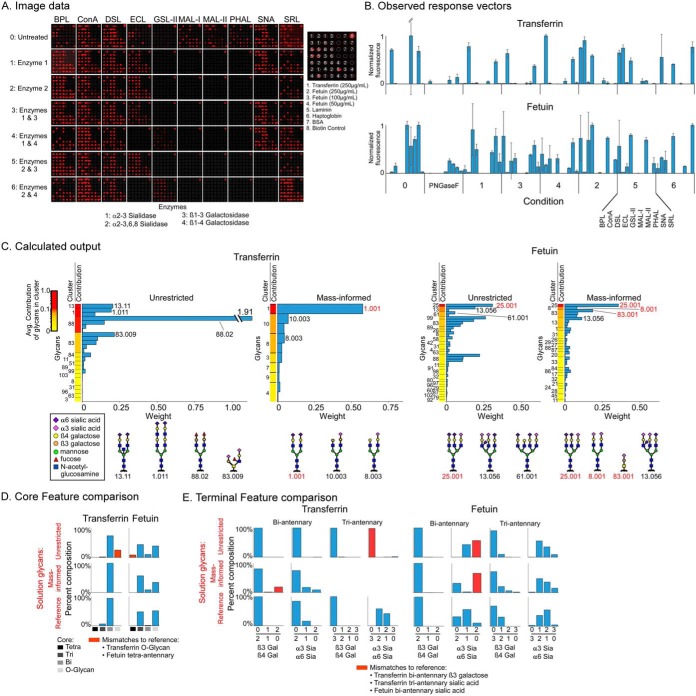
**Differentiating subterminal features on transferrin and fetuin.** (*A*) Raw image data. (*B*) Observed response vectors. The (*C*) solution glycans, (*D*) core feature summaries, and (*E*) terminal feature summaries are presented as in Fig. 3. The agreement between the solutions and the reference glycans is improved relative to the first experiment.

The experimental application of this sequence to transferrin and fetuin produced major changes in binding across conditions (Fig. 5*A*), consuming only 120 ng per protein (0.5 ng/array × 3 arrays/condition × 8 conditions × 10 lectins). The starting amount of material used to make the arrays was the same as above, about 16.6 μg. The observed response vectors were more complex than in the first experiment and showed great differences between transferrin and fetuin (Fig. 5*B* and Table S1). For transferrin, the solution glycans were more heavily weighted toward biantennary glycans with α2–6 sialylation on both arms (Fig. 5*C* and Table S1), in distinction to the first experiment. The unrestricted analysis mistakenly found biantennary glycans with bisecting GlcNAc (13.11) or doubly extended Galβ1–4GlcNAc (1.011), although both share the correct sialic acid and galactose linkages with the top reference glycan (1.001), which was found in the mass-informed analysis. For fetuin, the top hit (25.001) was the main triantennary glycan in the reference. The mass-informed analysis correctly found the top O-linked glycan (83.001) and a biantennary glycan (8.001) from the reference.

The core features (Fig. 5*D*) showed good agreement with the reference, with the mass-informed analysis eliminating O-glycan findings for transferrin and tetraantennary findings for fetuin. Likewise, the agreement of terminal features with the reference was much improved relative to the first experiment (Fig. 5*E*). The galactose linkages were accurately predicted for both proteins and both core types. The sialic acid linkages on transferrin were largely correct on the biantennary glycans, with increased accuracy using the mass-informed analysis (the minor amount of triantennary glycans was not detected by the mass-informed analysis). The sialic acid linkages on fetuin showed good agreement in the triantennary glycans, but some discrepancies in the biantennary glycans, potentially due to the low abundance of biantennary glycans—estimated to be 1.6% of the total (Fig. 2*B*). Therefore, the custom-designed sequence of lectin and exoglycosidases incubations improved the accuracy for probing specific, underlying features of glycosylation. Furthermore, the incorporation of information about masses further improved accuracy.

##### Using On-chip gmap to Assist Interpretation of MS Data

A useful application of this method could be to assist the interpretation of MS data. In basic analyses of detached glycans or glycopeptides, the masses of the glycans are identified, and in turn the monosaccharide compositions, but additional details about structure are not determined. The challenge is to determine the specific isomeric variants that comprise each mass. Because on-chip gmap is well suited to identifying specific linkages and motifs, we postulated that its information would be valuable in the interpretation of MS data.

**Fig. 6.**
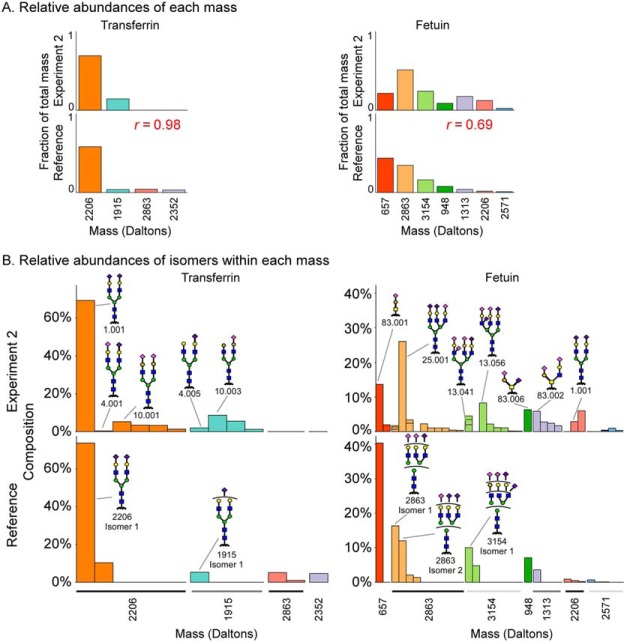
**Identifying component isomers of glycan compositions identified by MS.** (*A*) Using the solution glycans from the mass-informed analysis of experiment two, the weights of all isomers of a given mass were summed, and the sums were converted to their fractions of the total over all masses. Equivalent calculations were made for the reference glycans using their relative abundances from Fig. 2. (*B*) For each mass, the relative compositions of the isomers were compared between the solution glycans and the reference glycans. Divided columns in the solution glycans indicate proportions of specific isomers that were ambiguously defined in the reference glycans.

We first asked whether on-chip gmap produced relative proportions of the masses that generally matched known proportions, which would test the legitimacy of using the method to interpret abundances. Using the solutions from the mass-informed analysis of the second experiment (given in Fig. 5), we summed the weights of the glycans for each mass and then converted the sums to the fractions of the overall total. We calculated the equivalent fractions for the reference glycans and then compared the values. We found excellent agreement for both transferrin and fetuin, with overall correlations of 0.98 and 0.69, respectively (Fig. 6*A*). The 2,206 Da mass was dominant in both the solution and the reference glycans of transferrin, and the 1,915 Da glycan was much less abundant. Fetuin showed a broader distribution of glycans in both analyses. The solution and the reference agreed in the dominant glycans, with minor differences in rank.

The next question was whether on-chip gmap could help identify specific isomers that are most likely present for each mass. For each mass, we compared the isomers in the solution glycans to those in the reference glycans. For mass 2,206 Da of transferrin, the top prediction (glycan 1.001) matched the top reference isomer. On-chip gmap gave a number of the lesser isomers for 2,206 Da that overall match the abundance of the secondary isomer in the reference glycans. For mass 1,915 Da of transferrin, on-chip gmap found the top isomer in the reference (glycan 4.005) but among other isomers. Potentially the finding of glycan 10.003, which contains β1–3 galactose linkages, is correct because the galactose linkages in this mass were not carefully determined in the reference glycans.

For fetuin, five of the masses (657, 2,863, 3,154, 948, and 1,313 Da) had one or more matches in the top isomers. For mass 2,863 Da (glycan 25.001), the top prediction matched the second highest reference isomer, and it differed from the highest isomer by a single sialic acid linkage. Similarly, the top prediction for mass 3,154 Da (13.056) differs from the top reference isomer by a single galactose linkage. The relative heterogeneity within each mass also matched well. The uniformity of isomers for masses 657 Da and 948 Da, which have a single predominant isomer, contrasts the broad distribution of isomers for masses 2,863 Da and 3,154 Da.

The on-chip gmap results provided added detail: They gave the specific branch arrangements of the differing sialic acid and galactose linkages that were ambiguous in the reference set. Such information cannot yet be independently confirmed, but it provides leads for further study. Therefore, the combination of on-chip gmap and MS identified the proper distribution between masses, the correct level of heterogeneity within each mass, and the correct isomer for specific masses, especially the more-abundant ones. This analysis establishes that on-chip gmap can be valuable for assisting the interpretation of MS data.

## DISCUSSION

The on-chip gmap method takes advantage of the specificities of glycosidases and lectins, combined with glycan-array data and computational tools for integrating the information, to produce an approach for thoroughly investigating isomeric variants of glycans. As such, it could fill a valuable role in the analysis of protein glycosylation. Furthermore, it could reduce the amount of protein needed to get information about isomeric variants of glycans. Here, the method consumed just 120 ng of protein to get accurate information about the glycan isomers on fetuin and transferrin. More was used to create the arrays—about 16.6 μg in solution, in this embodiment—but most could be recovered for later use, and the amount could be lowered in various ways. One could use printing from just one pin, for example, or an ink-jet type of spotter that requires less material. Alternatively, if one has appropriate antibodies, the antibodies could be printed on the arrays and used to capture proteins out of solution. The incubation of a 1 μg/ml solution would typically saturate binding. Thus, based on the use of about 30–50 μl of solution, the protein requirement would be just 30–50 ng. But even if prepurification and direct spotting of the protein is necessary, the sample needs are favorable when contrasted with other approaches for probing isomeric variants. For example, an NMR study of glycoforms of cetuximab used about 2.5 mg of protein to assign isomers for the 10 most abundant structures (5); a previous study of the glycosylation of mucins purified from human serum required about 5 ml (21); the analysis presented here of detached N-glycans by MS/MS and fragmentation by MS^n^ used ∼300 μg of protein; and the analysis presented here by capillary electrophoresis used ∼50 μg of protein.

On-chip gmap method could be used in combination with other approaches for increased value. Clearly, the most useful combinations will be with MS analyses, owing to the complementary advantages and limitations of each method. Detached-glycan analysis provides the masses of intact glycans, but it provides no information on site occupancy and limited information on isomeric variants; on-chip gmap provides linkages and sequences but not masses or site occupancies, and glycopeptide analysis provides information on the attachment sites of the glycans on the protein backbone but presents challenges in identifying masses of complete, intact glycans and in finding all glycopeptides. For example, a study of the protein erythropoietin characterized site-specific heterogeneity in glycans but without assignments of linkages (10) and a study of glycoforms of monoclonal antibodies likewise did not make assignments of isomers (22). Therefore, the MS methods could be coupled to on-chip gmap to provide deeper information than possible by any single method. A practical strategy for accomplishing such experiments could be enriching proteins out of biological solutions using antibody arrays (11, 23), which may be compatible with both on-chip gmap and MALDI. The value of linking orthogonal methods has been appreciated previously, as in the use of antibody and lectin binding to interpret MALDI data (24) or in the combined use of NMR and MS (25), but automated algorithms to combine the methods were not available. The computational tools presented here may facilitate the linking of the data and the integration of additional, disparate technologies such as lectin arrays (26).

A limitation of on-chip gmap is its diminishing value for proteins with high heterogeneity or many similar glycans. The current method accounts for heterogeneity by deconvolving the signals from individual glycans, but the challenge gets more difficult as the glycoprotein becomes more complex. We show that, fundamentally, the method works, and that by increasing the experimental depth—more lectins and more rounds of enzymatic treatment—we get closer to the right answer even for a highly complex protein like fetuin. But for proper application, we must have output metrics that indicate the confidence of the findings and design algorithms to optimize the sequences of lectins and enzymes that probe specific glycans. The use of reference material that has been well characterized could help to clarify the limitations of complexity that are accessible with particular experiments and serve as a means of consistent calibration. In addition, heterogeneity could be addressed by fractionating glycoforms or domains prior to analysis.

A goal for further development is to increase the choices of lectins and enzymes. Many glycosidases and lectins already are available, and more are continually being discovered or developed. Not all of them function well for *in vitro* analyses, but it is likely that experimental optimization and protein engineering can improve activities in many cases. For example, researchers engineered a ricin-type lectin from earthworm to have high affinity to α2–6 sialic acid (27) and used directed evolution to produce specificity for 6-sulfated galactose (28). Another group created deletion mutants of the yeast peptide:N-glycanase enzyme to enhance deglycosylation activity (29). Anti-glycan antibodies are a good alternative and are being produced in increasing quantities (30), including antibodies generated from lamprey (31).

Another important goal for development is to increase the accuracy of the information about specificities. Glycan arrays facilitate detailed analyses of lectin specificity (32), and we recently developed their use for analyzing glycosidase specificity (16). The optimal use of glycan arrays will involve custom-produced arrays designed to probe specific areas of fine specificity. Researchers are increasingly creating arrays with specialized content, such as glucans that were purified from natural sources (24), human milk oligosaccharides (33), microbial glycans (34, 35), and various types of sialylated structures (36–38). Bead-based arrays may be particularly useful for rapid customization (39). In addition, new synthetic methods are enabling the production of structures that were previously difficult to synthesize, such as asymmetrically branched N-glycans (40, 41), which could reveal unusual fine specificities in lectins or glycosidases (16). Integrating glycan-array analyses with lectin structural information (42) also promises to increase precision in charactering specificities.

In conclusion, on-chip gmap with the integration of orthogonal data provides high-accuracy analyses of protein glycosylation, and it opens the door to detailed studies of glycosylation using small amounts of protein, such as would be available from clinical specimens. The direct analysis of the clinical specimens from patients could foster the discovery of glycoforms that are associated with disease. Once a glycan is discovered, a robust, highly reproducible assay could be developed that would be suitable for diagnostics. Furthermore, if researchers have the ability to thoroughly study protein glycosylation using tiny amounts of protein, they may be able to obtain new information about the features of glycans that affect biological mechanisms.

## DATA AVAILABILITY

The full raw data from the nanospray ionization-MS for the full glycan analysis and LTQ linear ion trap MS, including MS^n^ fragmentation for the sialic-acid linkage analysis, are available in the Supplemental Data.

## Supplementary Material

Table S1

## Footnotes

^1^ The abbreviations used are:

MS: mass spectrometry
gmap: glycan modification and probing
Gal: galactose
GlcNAc: N-acetyl-glucosamine.
