# Supplementary material for: Deciphering Protein Glycosylation by Computational Integration of On-chip Profiling, Glycan-array Data, and Mass Spectrometry
Source: Mol Cell Proteomics. 2018 Sep 26;18(1):28–40. doi: 10.1074/mcp.RA118.000906 (PMC6317472; doi:10.1074/mcp.RA118.000906)
Supplement: Table S1 [file RA118.000906_index.html]

Supplement to Deciphering protein glycosylation by computational integration of on-chip profiling, glycan-array data, and mass spectrometry | Molecular & Cellular Proteomics

## Supplemental Data

- Supplementary Tables - Tables S1, S2, S5, and S6
- Supplemental Data (to be published online) - Supplementary Methods Supplementary Tables Supplementary Figures
- Supplemental Data Files - Raw data files for the full FTMS spectra of N-glycans and MS/MS fragmentation spectra.
