# Supplementary material for: Deciphering Protein Glycosylation by Computational Integration of On-chip Profiling, Glycan-array Data, and Mass Spectrometry
Source: Mol Cell Proteomics. 2018 Sep 26;18(1):28–40. doi: 10.1074/mcp.RA118.000906 (PMC6317472; doi:10.1074/mcp.RA118.000906)
Supplement: Table S1 [file 138635_3_supp_209427_pfn6bh.pdf]

Supplemental Data

Deciphering protein glycosylation from small sample amounts by computational integration of on-chip profiling, glycan-array data, and mass spectrometry

Zachary Klamer, Peter Hsueh, David Ayala-Talavera, Brian Haab

Van Andel Research Institute, 333 Bostwick NE, Grand Rapids, MI 49503

Contents

Supplementary Methods

- Analysis of N-linked glycans on bovine fetuin

Supplementary Tables

- Table S1. Glycansolver output for unrestricted and mass informed analysis for transferrin and fetuin experiments 1 and 2 (see Excel file)
- Table S2. Partially ambiguous reference glycan sets for transferrin and fetuin with percent composition (see Excel file)
- Table S3. Summary of N-glycans observed in VARI and UGA fetuin
- Table S4. Percentages of β1-3 versus β1-4 linkage isomers determined by capillary electrophoresis
- Table S5. The normalized and raw experimental data for all glycoproteins in experiments 1 and 2 (see Excel file)
- Table S6. The unrestricted and mass restricted input lists for transferrin and fetuin respectively (see Excel file)

Supplementary Figures

- Figure S1. Full FTMS spectra of N-glycans observed in VARI and UGA fetuin samples
- Figure S2. MS/MS (CID) fragmentation of m/z 1216
- Figure S3. Capillary electrophoresis separations of a glucose ladder and separations of N-glycans from fetuin
- Figure S4. Evaluating fit quality

Supplementary Data

- Raw data files for the full FTMS spectra of N-glycans and MS/MS fragmentation spectra (separate zip file)

Supplementary Methods

Analysis of N-linked glycans on bovine fetuin

All analyses used purified bovine-fetuin obtained from a commercial source (F2379, Sigma-Aldrich). The methods are based on work presented previously (1). Approximately 300 μg of protein was used for combined MS analyses, and approximately 50 μg of protein was used for the electrophoresis analysis.

Release of N-linked glycans

The samples were reduced with 5 mM DTT for 45 minutes at 60˚C, alkylated with 15 mM IAA at room temperature for 30 minutes, reconstituted in a buffer of 50mM pH 7.6 Tris, and broken down into peptides and glycopeptides with Trypsin at 37˚C overnight.

Following protease digestion, the samples were cleaned with a C18 cartridge. SPE cartridges were cleaned with ~1 mL of MeOH, 1 mL of 100% ACN, 1 mL of 85% ACN in 0.1% formic acid, then 3 mL of 0.1% formic acid. The sample was applied, and then washed with 3 mL of 0.1% formic acid to remove salts. The peptides and glycopeptides were eluted from the column with 1 mL of 85% ACN in 0.1% formic acid, and dried by nitrogen. The N-glycans were released with PNGase F at 37˚C overnight. The released glycans were purified from the peptides by passing the digestion through another C18 cartridge, permethylated based on the methods of Anumula and Taylor (2), and then analyzed via NSI-FTMS/MS.

Per-O-methylation of N-linked glycans

The N-linked glycans were permethylated for structural characterization by mass spectrometry. Briefly, the dried eluate was dissolved with dimethyl sulfoxide and methylated with NaOH and methyl iodide. The reaction was quenched with water. The per-*O*-methylated carbohydrates were extracted with methylene chloride and dried under N_2.

Profiling and Fragmentation analysis by Nanospray Ionization – Mass Spectrometry (NSI-FTMS/MS)

The permethylated glycans were reconstituted in 50% MeOH/1 mM NaOH and introduced to the mass spectrometer (Thermo Fusion Tribrid Orbitrap) with direct infusion at a flow rate of 0.5 μL/min and at a resolution of 120,000. Full MS spectra as well as an automated “TopN” MS/MS program of the top 300 peaks were collected and fragmented with collision-induced fragmentation (CID) at an energy percentage of 35%.

Potential N-glycans were determined based on full mass by using online tool Glycomod. Full and MS/MS spectra were annotated with the help of Glycoworkbench v2.1.

Analysis by LTQ Linear Ion Trap Mass Spectrometry (LTQ MS) including MSn fragmentation

The permethylated glycans were diluted into 1 mM lithium carbonate/50% MeOH and infused directly into an LTQ Orbitrap Discovery Mass Spectrometer (ThermoScientific) at a flow rate of 0.5 mL/min for nanospray ionization (NSI). A full FTMS spectrum was obtained at a 30,000 resolution for each sample to determine which glycans in the samples contained sialic acids.

Once the sialic-acid-containing glycans were identified, they were subjected to MS^n analysis designed to determine whether the sialic attachments in each glycan were α2,3 or 2,6 linked. The method, first published by Anthony *et al.* (3), subjects the sample to multiple ion-selection and fragmentation steps within the ion trap to break down a complex-type glycan to a single galactose and then observing the fragmentation pattern.

N-glycan labeling by APTS and analysis by Capillary Electrophoresis.

Released N-glycans were also analyzed by capillary electrophoresis to determine the relative amounts of β1-3 to β1-4 linked galactose in the triantennary glycoform.

Because high sialyation could obscure the results, the released N-glycans were desialyated with a general neuraminidase (New England Biolabs) overnight at 37˚C. The

released glycans were then labeled with APTS (8-aminopyrene-1,3,6-trisulfonic acid) in a solution containing 0.5 M sodium cyanoborohydrate (Fluka) and 0.2 M APTS (ABSciex). The labeled glycans were diluted with mΩ water before analysis with capillary electrophoresis. A ladder of neutral dextrans was also separated as a control and size calibrator.

Capillary electrophoresis was carried out on a ABSciex PA-80 system using a PVA-neutral-coating capillary (60.2 cm total length, 50 cm to the window). The separation buffer was a linear-acrylamide carbohydrate separation buffer (ABSciex), and detection was carried out with an attached Ar-ion laser system (λex 488nm, λem 520nm). The sample was briefly injected (0.5 psi, 6 sec), and it was separated for 30 minutes with a 25 kV applied voltage in reversed mode.

Supplementary Tables

Table S3. Summary of N-glycans observed in VARI and UGA fetuin.

				%Area VARI	%Area UGA
1579.78		$(Man)_2$+ $(Man)_3(GlcNAc)_2$	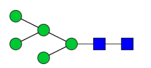	0.5%	0.1%
2792.38		(Hex)2 (HexNAc)2 (NeuAc)2 +(Man)3(GlcNAc)2	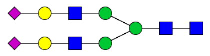	3.1%	4.5%
3037.51		$(Hex)_2$ $(HexNAc)_3$ $(NeuAc)_2$ + $(Man)_3(GlcNAc)_2$	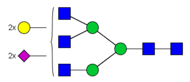	0.8%	0.2%
3241.61		(Hex)3 (HexNAc)3 (NeuAc)2 +(Man)3(GlcNAc)2	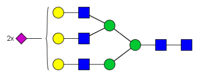	1.9%	1.5%
3153.56		$(Hex)_2$ $(HexNAc)_2$ $(NeuAc)_3$ + $(Man)_3(GlcNAc)_2$	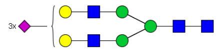	0.9%	0.6%
3602.78		$(Hex)_3$ $(HexNAc)_3$ $(NeuAc)_3$ + $(Man)_3(GlcNAc)_2$	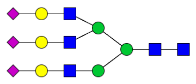	62.9%	69.3%
3963.96		$(Hex)_3$ $(HexNAc)_3$ $(NeuAc)_4$ + $(Man)_3(GlcNAc)_2$	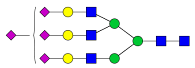	29.2%	23.3%
4413.18		$(Hex)_4$ $(HexNAc)_4$ $(NeuAc)_4$ + $(Man)_3(GlcNAc)_2$	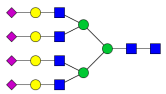	0.5%	0.4%
4774.36		$(Hex)_4$ $(HexNAc)_4$ $(NeuAc)_5$ + $(Man)_3(GlcNAc)_2$	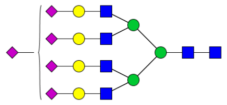	0.1%	0.1%

Table S4. Percentages of β1-3 versus β1-4 linkage isomers determined by capillary electrophoresis. Galactose with a black line corresponds to the β1-3 linkage isomer.

Glycoform	Area	Percentage
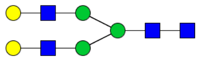	1950	12.4
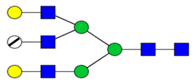	1350	8.6
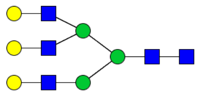	12440	79.0

Supplementary Figures

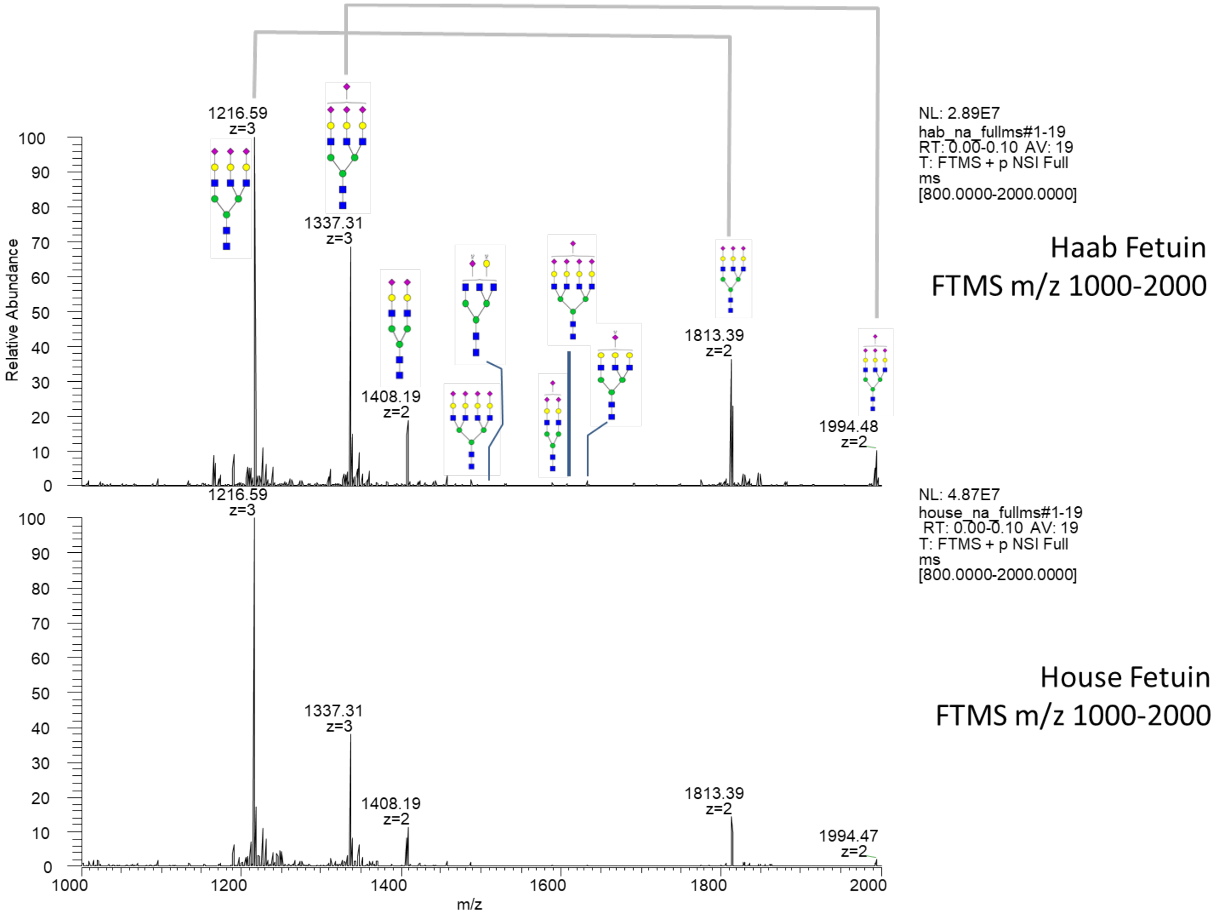

Figure S1. Full FTMS spectra of N-glycans observed in VARI (labeled 'Haab Fetuin) and University of Georgia (labeled 'House Fetuin') bovine fetuin samples. The top spectrum was acquired for this project using fetuin provided for this project (F2379, Sigma), and the bottom spectrum is previous data from the University of Georgia using fetuin previously acquired (F3004, Sigma).

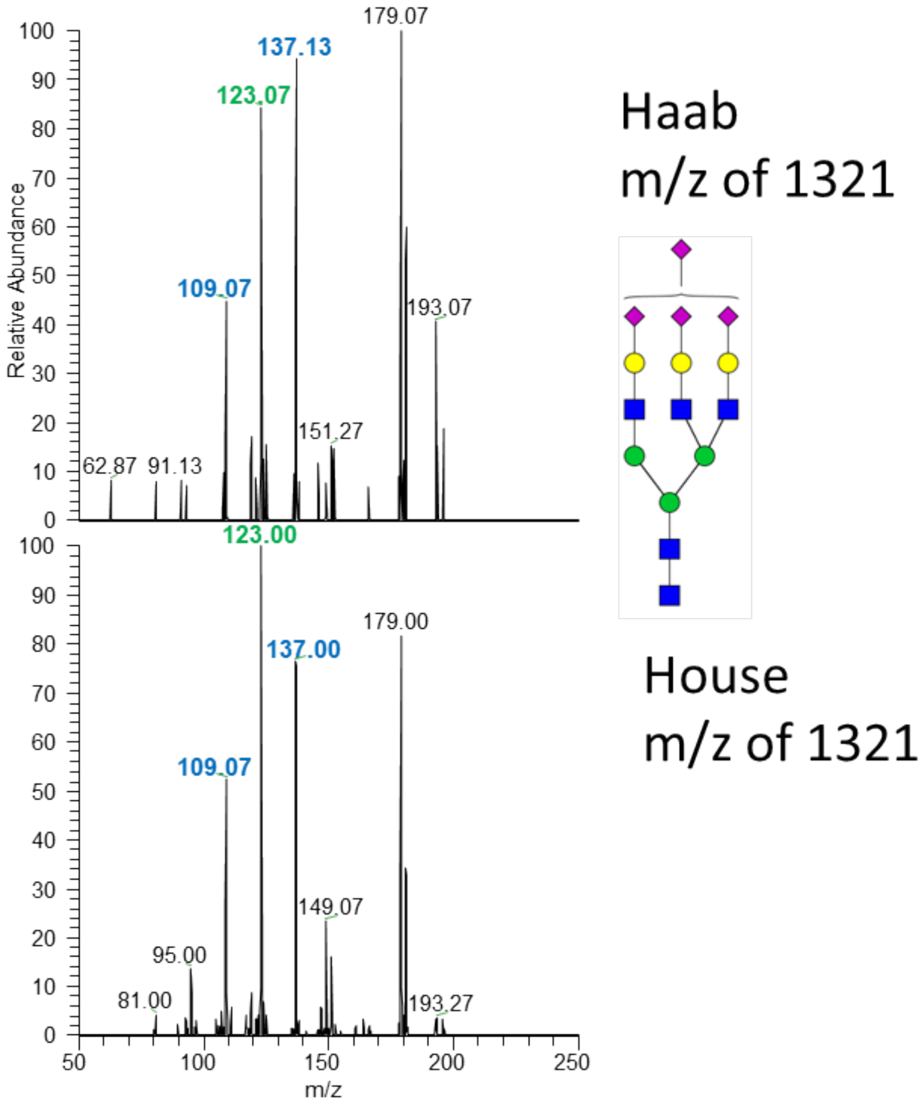

Figure S2. Representation MS/MS (CID) fragmentation of a terminal galactose to determine sialic-acid attachment sites. MSn fragmentation analysis was used to break down the glycan, then isolate and subsequent fragment down to the galactose. Cross-ring fragmentation was then used to determine if the sialic acid attached to that galactose was 2,3- or 2,6-linked. Fragments corresponding to a 2,3 linkage are labeled in blue, while 2,6 linkages are labeled in green. The top spectrum (labeled ‘Haab’) was acquired for this project using fetuin provided for this project (F2379, Sigma), and the bottom spectrum (labeled ‘House’) is previous data from the University of Georgia using fetuin previously acquired (F3004, Sigma). These results show that the glycan contains some combination of 2-3, and 2-6 linked sialic acid, and that it is very similar to the house-provided fetuin.

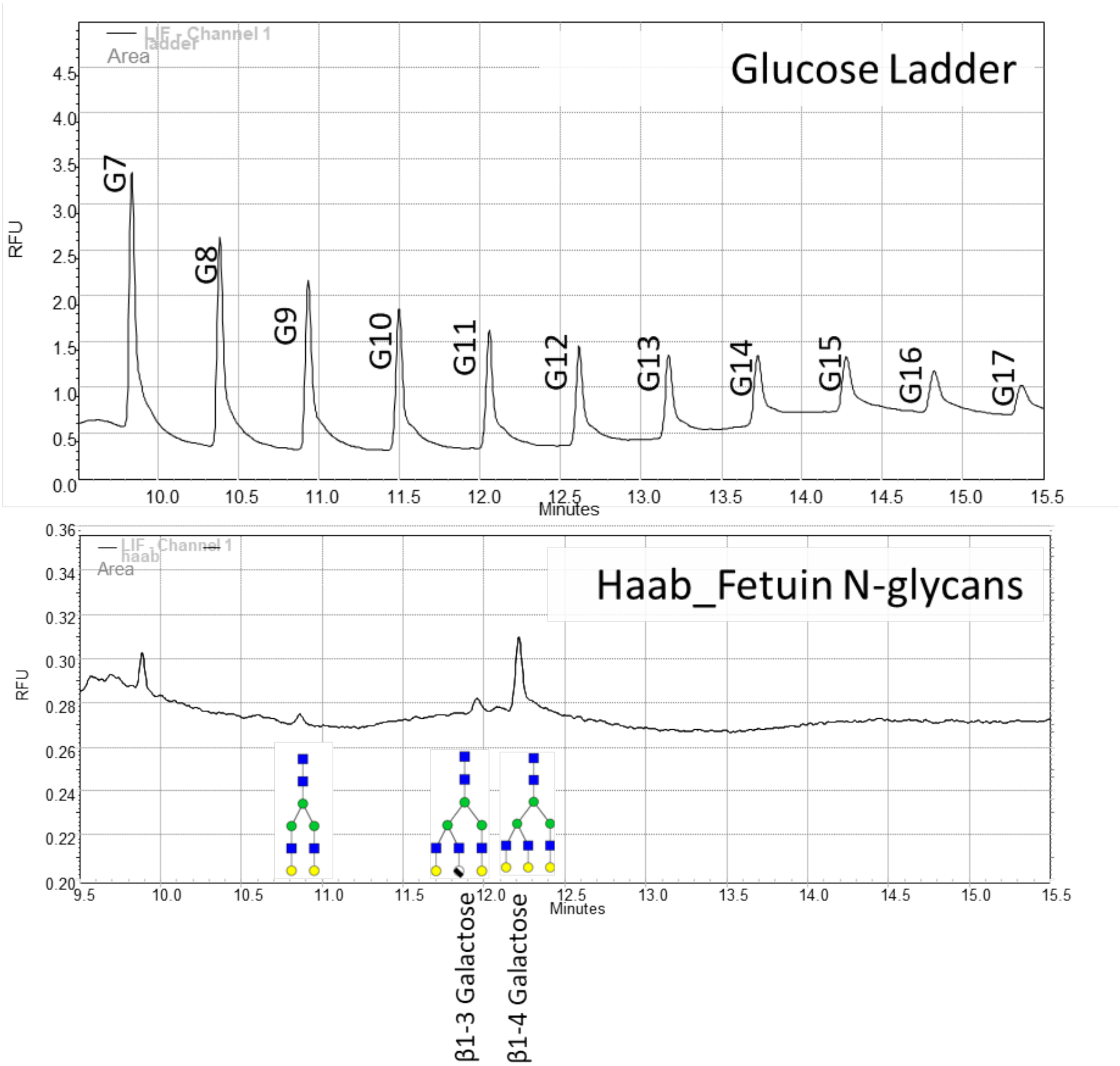

Figure S3. Capillary electrophoresis separations of a glucose ladder (top) and separations of N-glycans from fetuin. The top panel presents an APTS-labeled glucose ladder used to calibrate the migration times for each glycoform. The bottom panel shows the migration of the major glycoforms (minus sialic acids) of fetuin. The three major glycoforms migrate as expected from previous data, with the β1-3 and β1-4 separating enough to provide baseline separation from each other. The areas and relative percentages of each glycan are shown in Table S2 and are in agreement with literature values for these glycoforms, such as those tabulated by Green, et al. (4).

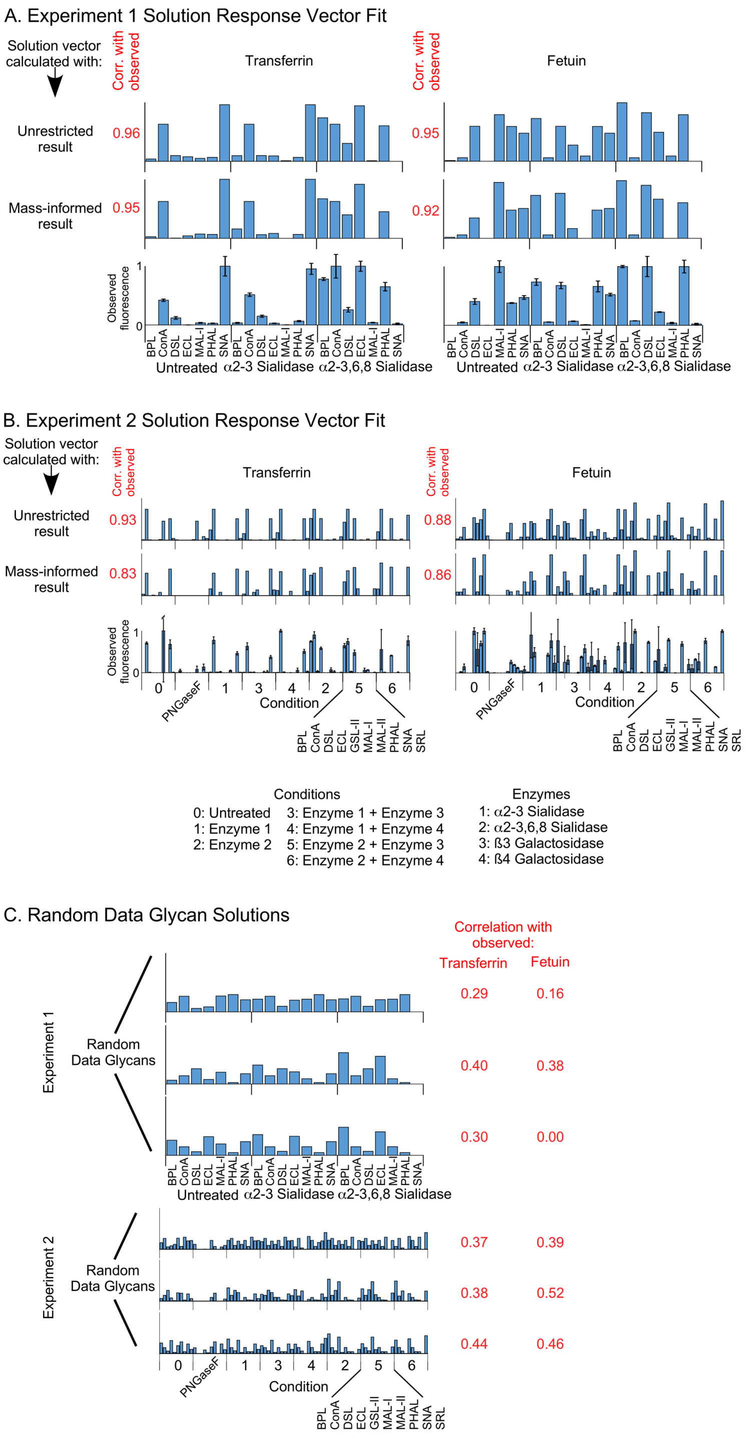

Figure S4. Evaluating fit quality. The solution response vectors of the glycans from A) Experiment 1 and B) Experiment 2 were multiplied by their weights and summed to arrive at a solution response vector. C) Three random solution response vectors were constructed by the random shuffling of the observed response vectors from Experiment 1 and Experiment 2. The random vectors were used in the same way as the true observed vector. The correlations between the resulting solution response vectors and the observed response vector are much lower than between the reference-glycan solution response vector and the observed response vector.

References

1. Shajahan, A., Heiss, C., Ishihara, M., and Azadi, P. (2017) Glycomic and glycoproteomic analysis of glycoproteins-a tutorial. *Anal Bioanal Chem* 409, 4483-4505.
2. Anumula, K. R., and Taylor, P. B. (1992) A comprehensive procedure for preparation of partially methylated alditol acetates from glycoprotein carbohydrates. *Anal Biochem* 203, 101-108.
3. Anthony, R. M., Nimmerjahn, F., Ashline, D. J., Reinhold, V. N., Paulson, J. C., and Ravetch, J. V. (2008) Recapitulation of ivig anti-inflammatory activity with a recombinant igg fc. *Science* 320, 373-376.
4. Green, E. D., Adelt, G., Baenziger, J. U., Wilson, S., and Van Halbeek, H. (1988) The asparagine-linked oligosaccharides on bovine fetuin. Structural analysis of n-glycanase-released oligosaccharides by 500-megahertz 1h nmr spectroscopy. *J Biol Chem* 263, 18253-18268.
